# Similarity in milk microbiota in replicates

**DOI:** 10.1002/mbo3.1383

**Published:** 2023-09-30

**Authors:** Josef Dahlberg, Erik Pelve, Johan Dicksved

**Affiliations:** ^1^ Department of Clinical Sciences Swedish University of Agricultural Sciences Uppsala Sweden; ^2^ Department of Anatomy, Physiology and Biochemistry Swedish University of Agricultural Sciences Uppsala Sweden; ^3^ Department of Animal Nutrition and Management Swedish University of Agricultural Sciences Uppsala Sweden

**Keywords:** microbiota, milk, mock community, repeatability, reproducibility

## Abstract

Receiving the same results from repeated analysis of the same sample is a basic principle in science. The inability to reproduce previously published results has led to discussions of a reproducibility crisis within science. For studies of microbial communities, the problem of reproducibility is more pronounced and has, in some fields, led to a discussion on the very existence of a constantly present microbiota. In this study, DNA from 44 bovine milk samples were extracted twice and the V3–V4 region of the 16S rRNA gene was sequenced in two separate runs. The FASTQ files from the two data sets were run through the same bioinformatics pipeline using the same settings and results from the two data sets were compared. Milk samples collected maximally 2 h apart were used as replicates and permitted comparisons to be made within the same run. Results show a significant difference in species richness between the two sequencing runs although Shannon and Simpson's diversity was the same. Multivariate analyses of all samples demonstrate that the sequencing run was a driver for variation. Direct comparison of similarity between samples and sequencing run showed an average similarity of 42%–45% depending on whether binary or abundance‐based similarity indices were used. Within‐run comparisons of milk samples collected maximally 2 h apart showed an average similarity of 39%–47% depending on the similarity index used and that similarity differed significantly between runs. We conclude that repeated DNA extraction and sequencing significantly can affect the results of a low microbial biomass microbiota study.

## Graphical abstract

For this study, bacterial DNA from 44 milk samples was extracted twice, and the V3–V4 region of the 16S rRNA gene was sequenced in two separate runs. After submitting the sequence data to the same bioinformatic pipeline, results from the two runs are compared. We show that repeated DNA extraction and sequencing can significantly affect the results of a low microbial biomass microbiota study.

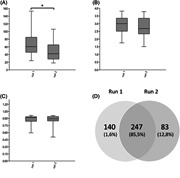

## 1 BACKGROUND

The ability to get the same, or similar, results from repeated experiments is one of the pillars that science is founded on. Technical replicates, that is, repeated measurements from the same sample, are used to show variation or similarity between measuring equipment and protocols, while biological replicates generally are defined as measurements of biological distinct samples that show biological variation.

In the past decade, sequencing of amplicons generated from the 16S rRNA gene has become the most commonly used technique to describe bacterial communities in various environments. A technical challenge with 16S amplicon‐sequencing is that the technique is prone to the introduction of biases (see Pollock et al., 2018 or Nearing et al., 2021, for review), results can vary with the type of primers used (Clooney et al., 2016) and the bioinformatics can substantially affect the results (O'Sullivan et al., 2021). Laboratory and reagent contamination has been shown to largely affect the results in microbiota studies where the bacterial biomass is low (Dahlberg et al., 2019; de Goffau et al., 2019; Lauder et al., 2016; Salter et al., 2014). As a consequence, methods to identify and cure data from contamination have been developed (Alipour et al., 2018; Davis et al., 2018; Karstens et al., 2019; Łukasik et al., 2017). The ability to get the same results from sample replicates has been examined (Kennedy et al., 2014; Marotz et al., 2019; Schwenker et al., 2022; Wen et al., 2017) and it has been shown that samples with low bacterial biomass have a lower reproducibility (Kennedy et al., 2014). Together these factors contribute to the reported low repeatability and reproducibility in microbiota studies.

In this study, we describe the similarity in microbiota composition between replicates of the same milk samples (prepared with similar but not identical lab protocols) from two separate 16S rRNA gene sequencing runs, further referred to as type 1 replicates. A bacterial‐based mock community was included as a control and compared in the same manner. We also compared milk samples collected from the same quarter and within a short time frame in the same sequencing run, referred to as type 2 replicates. The results from this study add to the list of biases that can affect the results of studies of microbiota, in general, and milk in particular.

## 2 METHODS

**Figure 1:**
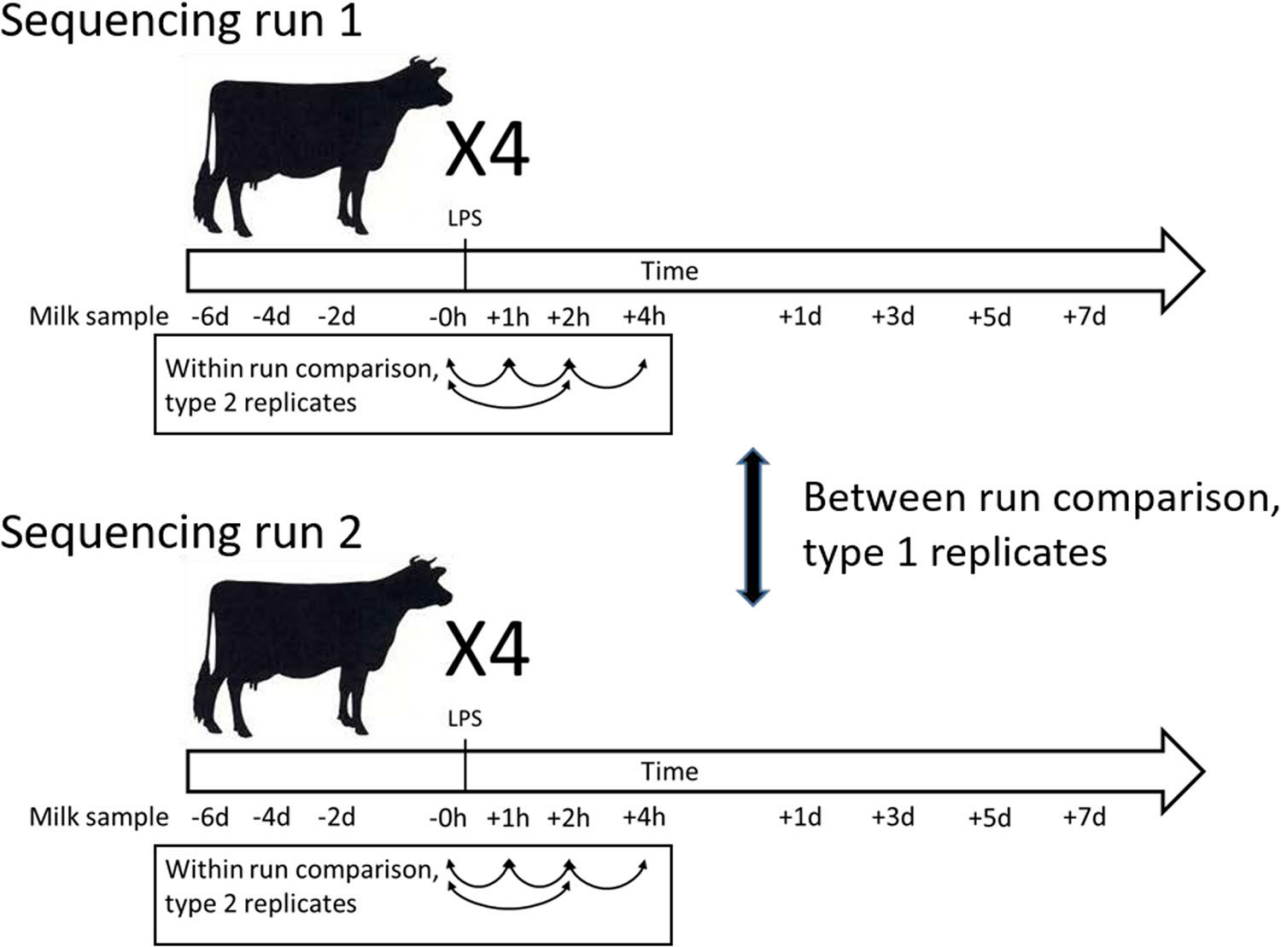
Schematic illustration of study design. Four lactating dairy cows were infused with *E. coli* lipopolysaccharide (LPS) in one udder quarter; milk samples were collected before and after infusion as indicated. Aliquots of milk samples were subjected to DNA extraction, sample processing, and sequencing in two separate runs. The similarity in microbiota was compared between runs (i.e., type 1 replicates) and for selected samples within the same run (i.e., type 2 replicates).

Milk samples came from an experiment that examined the effect of lipopolysaccharide (LPS) infusion into the mammary gland of healthy lactating dairy cows, previously described by Johnzon et al. (2018) and Dahlberg et al. (2023). In short, nine healthy lactating dairy cows were infused with LPS in one quarter and the inflammation processes were followed through repeated blood and milk sampling. Equally many cows were infused with sterile saline for comparison. Eleven milk samples were taken from each infused quarter during the experiment; from each sampling point, 5 mL of milk was aliquoted and stored until DNA extraction. Forty‐four milk samples from four LPS‐infused cows were subjected to DNA extraction and sequencing in two separate runs. A schematic illustration of the study layout is shown in Figure 1.

### 2.1 DNA preparation and sequencing run 1

Sample processing for sequencing run 1 has been described by Dahlberg et al. (2023), for clarity and comparison between sequencing runs; 1 mL of room temperature, well‐mixed milk was used for DNA extraction. After centrifugation (at 13,000*g*, 5 min), DNA was extracted from the cell pellet and fat layer using the DNeasy PowerFood Microbial kit (Qiagen; Lot no. 157017245); bead beating (Precellys24; Bertin Technologies; 2 × 45 s at 6500 rpm with 1 min pause) was included for cell lysis. For each round of DNA extraction, an empty vial into which the first reagent was added was used as a no‐template DNA extraction control (NTC) and processed as the milk samples. A bacterial mock community was included for method evaluation as previously described (Dahlberg et al., 2019). The mock community was created with equal numbers of cells and prepared in two different dilutions (10^7^ and 10^5^ cells per mL of each bacterial species) and DNA was extracted and amplified in duplicates using the same protocol as the milk samples.

Extracted DNA from milk samples, NTCs, and the bacterial mock communities were subjected to polymerase chain reaction (PCR) amplification and used to prepare an Illumina MiSeq sequencing library in a two‐step manner with primers previously described by Hugerth et al. (2014). In the first step, the V3–V4 region of the 16S rRNA gene was amplified, and in the second step, specific barcodes and Illumina adaptors were attached. One barcode combination per sample (i.e., milk, NTC, and bacterial‐based mock community samples) was used. DreamTaq PCR master mix (2×) (Thermo Fisher Scientific) was used in both PCR reactions, in the first PCR reaction 35 cycles were run with 3 µL of DNA as a template, and in the second 10 cycles and 5 µL of DNA was used. PCR products from the first and second PCR reactions were purified with Ampure Beads (Beckman Coulter) before continuation, 0.9 and 0.7 volumes of beads per volume of PCR product in the first and second PCR, respectively. All DNA extraction and first PCR preparations were performed in a laminar air‐flow hood cleaned with 10% bleach, 70% ethanol, and UV‐irradiated for 30 min before execution. After the second PCR and cleaning, DNA was quantified with a Qubit 3.0 Fluorometer (Life Technologies) and thereafter pooled into equimolar amounts. The DNA pool was concentrated using Ampure Beads and elution in a smaller volume and thereafter cleaned through gel extraction (GeneJET, Gel Extraction Kit; Thermo Fisher Scientific) to ensure DNA strand length. Sequencing was performed on an Illumina MiSeq sequencer with v3 sequencing chemistry, 2 × 300 bp with 10% PhiX (Illumina Inc.) at the Science for Life Laboratory.

### 2.2 DNA preparation and sequencing run 2

For the second sequencing run, DNA was prepared as follows: 1 mL of milk was centrifuged at 13,000*g* for 5 min; the supernatant and the fat layer were removed and DNA was extracted from the cell pellet using the PowerFood Microbial DNA isolation kit (MO BIO Laboratories Inc., Lot no PF14F2 later renamed to Qiagen DNeasy PowerFood Microbial kit). DNA extraction was performed according to the manufacturer's instructions except that Precellys24 (Bertin Technologies, 2 × 45 s at 6500 rpm with 1 min paus) was used for cell lysis. A blank sample with no starting material was included for each round of DNA extraction; these NTCs were PCR‐amplified as the milk samples and barcoded with one barcode combination. The same bacterial‐based mock community as previously described was included in two different dilution levels (10^7^ and 10^5^ cells per mL of each bacterial species). DNA extraction and first PCR preparations were performed in a laminar air‐flow hood cleaned with 10% bleach, 70% ethanol, and UV‐irradiated for 30 min before execution. PCR amplification and attachment of barcodes were performed same as for sequencing run 1 with the following exceptions: (a) Phusion high‐fidelity polymerase (Life Technologies) was used, (b) 0.5 or 5 µL of DNA was used as template in the first PCR, (c) 10 µL of DNA was used as template in second PCR, (d) 0.8 volumes of Ampure beads were used for DNA cleaning, and (e) no gel extraction was performed. Thermocycling conditions were: initial denaturation at 98°C for 30 s, thereafter denaturation at 98°C for 10 s, annealing at 60°C for 30 s, and elongation at 72°C for 7 s, and final elongation at 72°C for 2 min. Thirty‐five cycles were run in the first PCR creating amplicons and 10 cycles were run in the second PCR. Samples were pooled into equimolar amounts and sequenced on an Illumina MiSeq sequencer with v3 sequencing chemistry (Illumina Inc.) at the Science for Life Laboratory.

### 2.3 Illumina sequence data analysis

Data from the two sequencing runs were processed in parallel with the same settings as previously described (Iversen et al., 2022). In short, the DADA2 pipeline was used to denoise, dereplicate reads, merge pair‐end reads, and remove chimeras from the raw demultiplexed reads (Callahan et al., 2016). Amplicon sequence variants (ASVs) were assigned to reference sequences using the assignTaxonomy command (Wang et al., 2007) against the 132 release of the SILVA rRNA database (Quast et al., 2013), formatted for DADA2 by B. Callahan (https://benjjneb.github.io/dada2/training.html, accessed on 19 October 2019). The Phyloseq R package was used to construct ASV frequency tables for subsequent statistical analysis (McMurdie & Holmes, 2013). The data analysis was performed and alpha diversity was calculated on relative abundance data at the genus level. Descriptive analysis on sequencing results, statistical calculations, and multivariate analyses (Principal Coordinates Analysis [PCoA's]) were performed using Microsoft Excel 2016, Paleontological Statistics program (PAST, ver 4.05) (Hammer et al., 2001) and R (ver 3.5.3) (R Core Team, 2019). *T*‐test was used when two groups were compared and for statistical analysis, values are presented as mean ± standard deviation. For statistical analysis of the PCoA, an analysis of similarity (ANOSIM) was used. Statistical significance was set at the level *p* < 0.05.

### 2.4 Data analysis

The data set contained in total of 3.4 million reads, the number of reads per sample was higher in sequencing run 1 (56,930 ± 17,496) compared to run 2 (19,955 ± 11,104). Data from the 44 milk samples included in sequencing run 1 and 2 were merged in R. The merged data set was used to analyze similarities and differences between the separate sequence runs. For type 1 replicates, the clustering of samples was analyzed by PCoA and ANOSIM using Bray Curtis and Dice similarity indexes. Two similarity matrices, based on the same mentioned similarity indexes, were created in PAST. The similarity of the type 1 replicates between sequencing runs was then measured directly from the similarity matrix. Replicates of the bacterial mock community from sequencing run 1 and 2 were also merged into the data set and compared for the direct similarity between runs.

Sample similarity within the sequencing run was measured from a similarity matrix in a comparable manner as for between runs. The two similarity matrices, based on Bray Curtis and Dice similarity indexes created in PAST were used. The relevant comparisons were samples collected from the same individual (udder quarter) 1–2 h apart. More specifically: 1 h postintervention was compared to 0 h before the intervention, 2 h postintervention was compared to 0 h before intervention, 2 h postintervention was compared to 1 h postintervention, and 4 h postintervention was compared to 2 h postintervention. These samples were biological replicates but were simultaneously expected to have a high similarity in their bacterial composition (further elaborated in Section 4).

## 3 RESULTS

### 3.1 Difference between runs

**Figure 2:**
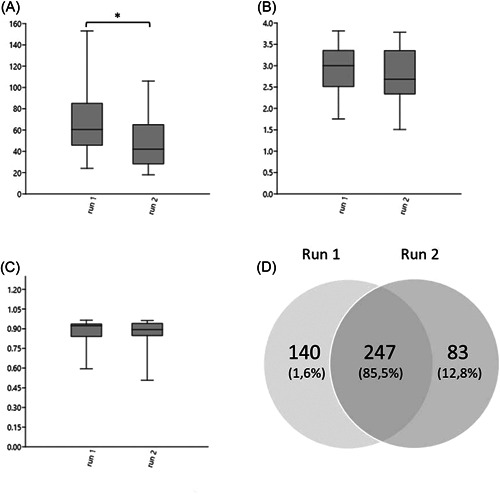
Boxplots of bacterial richness and diversity in type 1 replicate milk samples in different runs. (A) Chao‐1 richness. (B) Shannon diversity. (C) Simpson diversity. Brackets with stars above boxes indicate that they differ significantly. For boxes, the 25%–75% quartiles are in the box, and max and min are shown as whiskers. (D) Venn diagram showing the number of shared genera within two sequencing runs of the same 44 samples. The number within parenthesis represents the proportion of reads within each group.

The 44 milk samples that were subjected to DNA extraction and sequencing twice contained in total of 470 different bacterial taxa. The milk samples sequenced in run 1 had significantly more bacterial taxa, at the genus level, per sample (Chao‐1) compared to run 2 (65.7 ± 29.1 vs. 48.3 ± 24.7, *p* < 0.01 *t*‐test) while there was no difference in Shannon or Simpson diversity (Figure 2). A total of 247 bacterial taxa were shared between the two sequencing runs, 140 bacterial taxa were only identified in run 1, and 83 bacterial taxa were only identified in run 2 (Figure 2). The accumulated data (i.e., reads) from bacterial taxa that were shared between run 1 and 2 constituted a larger proportion of the data in run 1 compared to run 2 (Figure 2).

### 3.2 Microbiota similarities between runs

**Figure 3:**
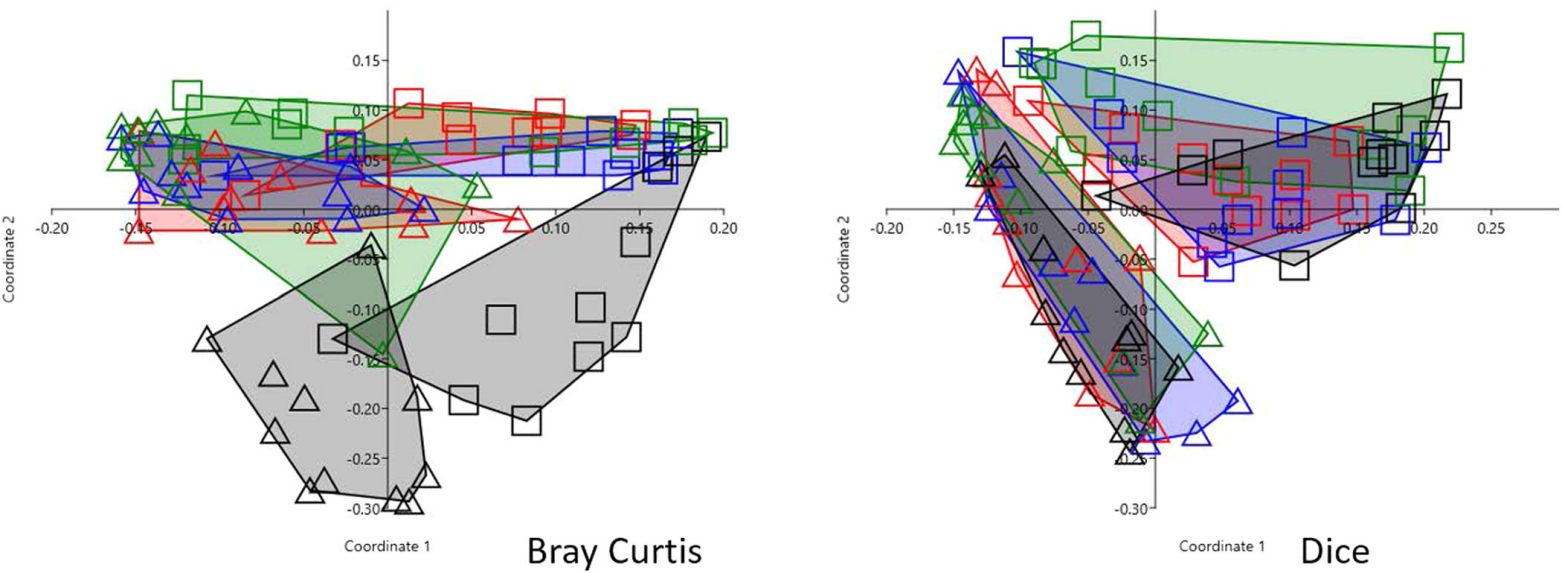
PCoA with 44 milk samples processed and sequenced twice, type 1 replicates. Samples are grouped by individual (color) and sequencing run (symbol). The similarity index used is indicated in the figure. For three out of four individuals, there were significant differences between runs, when analyzed in ANOSIM, for both similarity indexes used.

**Figure A1:**
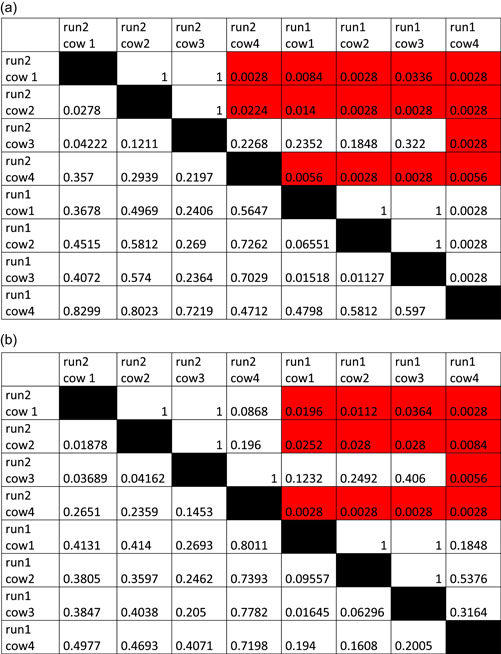
Analysis of similarities (ANOSIM) with (A) Bray Curtis and (B) Dice similarity index for 44 milk samples processed and sequenced twice, type 1 replicates. A PCoA of the same data is presented in Figure 3 in the main text. For both tables, Bonferroni corrected *p*‐values are presented above the diagonal and the *R* values below the diagonal.

The similarity between sequence runs was explored by PCoA, ANOSIM, and direct comparison of similarity between technical replicates from a similarity matrix. The PCoA revealed that the sequencing run had a bigger effect on the microbiota composition than the individual cow (Figure 3). The difference between sequencing runs was more pronounced when the Dice similarity index was applied to the data compared to the Bray‐Curtis similarity index (Figure 3). Regardless of the similarity index used, the ANOSIM confirmed that the difference between the runs was significant (Bonferroni‐corrected *p* < 0.01). For three out of four individuals, there were significant differences between runs, when analyzed in ANOSIM, for both similarity indices used (Appendix Figure A1).

**Figure 4:**
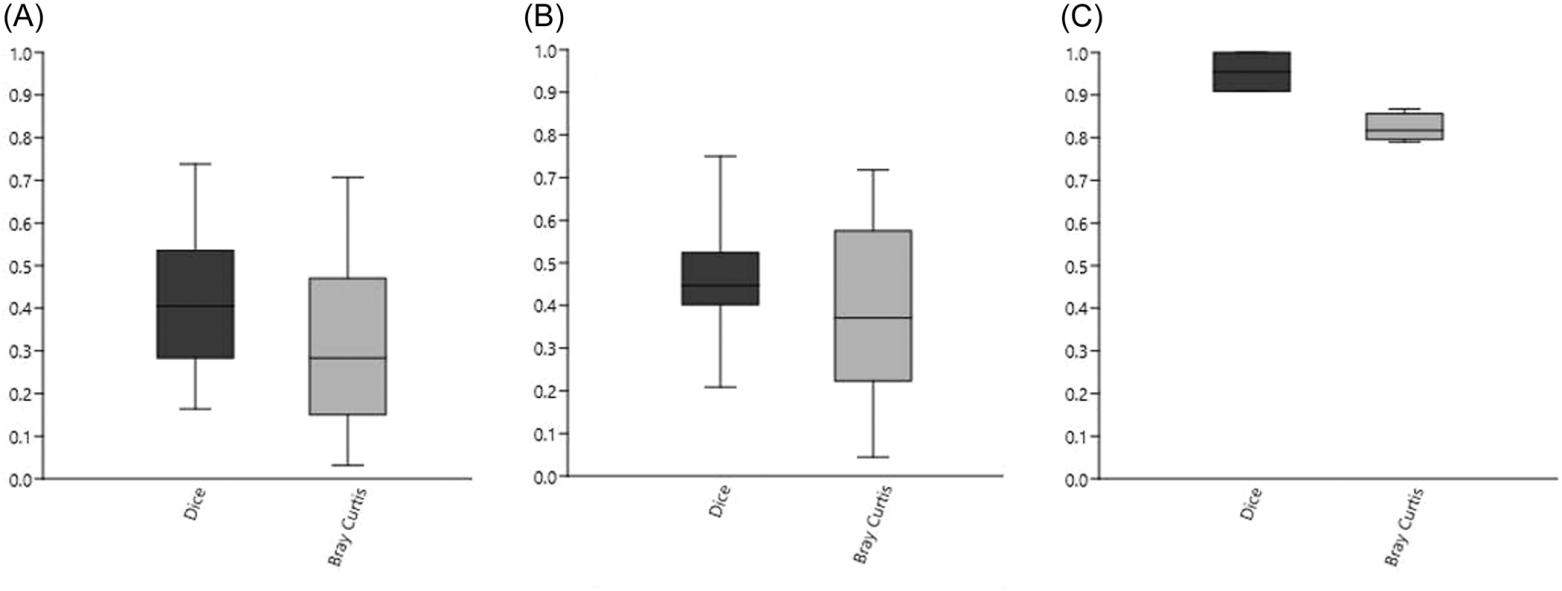
Boxplots of direct comparison of similarity in type 1 and type 2 replicates using different similarity indexes. (A) Type 1 replicates of milk samples sequenced in two different runs (*n* = 44). (B) Type 2 replicates of milk samples (taken 1–2 h apart) sequenced within the same run (*n* = 32). (C) Type 1 replicates of a bacterial mock community sequenced in two different runs (*n* = 4). For boxes, the 25%–75% quartiles are in the box, and max and min are shown as whiskers.

When direct comparisons of the similarity of type 1 replicates of milk samples from the two sequencing runs were analyzed the average similarity varied with the similarity index used. For the Dice similarity index, the average similarity was 0.42 ± 0.16, and for the Bray‐Curtis similarity index, the average similarity was 0.32 ± 0.18, Figure 4A. In comparison, the average similarity of type 1 replicates of the bacterial mock community was 0.95 ± 0.05 for the Dice similarity index and 0.82 ± 0.03 for the Bray Curtis similarity (Figure 4C).

### 3.3 Microbiota similarity within runs

The similarity between type 2 replicates, samples that were taken 1–2 h apart from the same individual (udder quarter), were analyzed in the data set. The average Dice similarity between samples taken 1–2 h apart was 0.47 ± 0.12 and the average Bray Curtis similarity was 0.39 ± 0.19 (Figure 4B).

**Figure 5:**
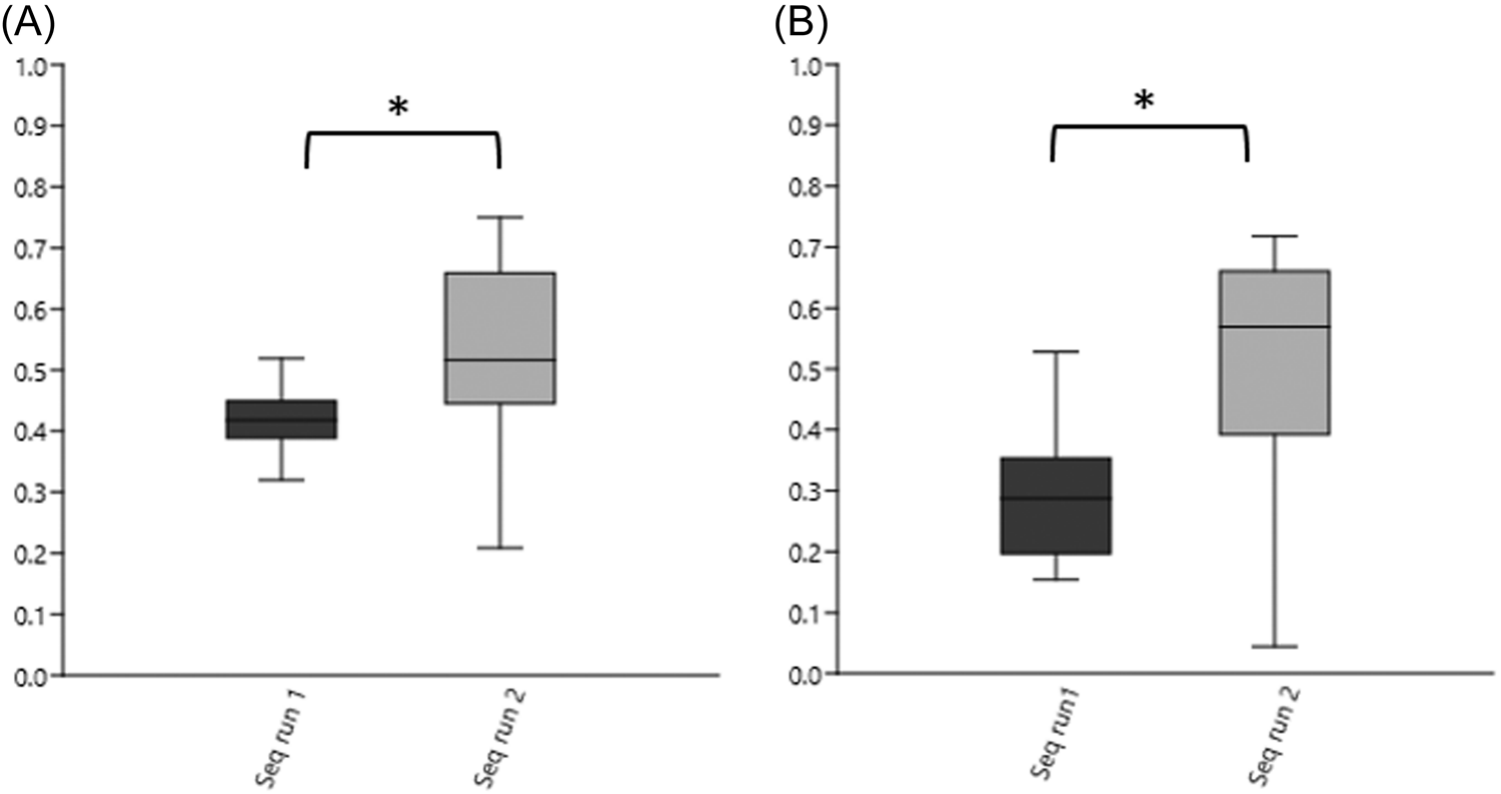
Boxplots of direct comparison of similarity for type 2 replicates separated by sequencing run. (A) Dice similarity index. (B) Bray Curtis similarity index. Number of comparisons within each run = 16. Brackets with stars above boxes indicate that they differ significantly in the *t*‐test. For boxes, the 25%–75% quartiles are in the box, and max and min are shown as whiskers.

As we noted that the similarity between milk samples were comparable regardless if the comparison was between type 1 or type 2 replicates (as seen in Figure 4A,B), this phenomenon was further explored. For both similarity indices used, there was a significantly higher similarity between type 2 replicates in sequencing run 2 compared to sequencing run 1 (Figure 5) (*p* < 0.05, *t*‐test).

## 4 DISCUSSION

In this text, the term type 1 replicates is used when comparing milk samples between different sequencing runs as the microbiota in identical samples were analyzed twice. However, some variations in the DNA extraction and PCR amplification existed between the two sequencing runs. Even though we used the same DNA extraction kit (MoBio PowerFood changed its name to DNeasy PowerFood after Qiagens purchased MoBio Laboratories), the batch number has been shown to influence results in microbiota studies (de Goffau et al. 2018, 2019; Salter et al., 2014). Another difference between the sequencing runs was the inclusion of the milk fat layer in sequencing run 1. It has previously been shown that milk fraction (whole milk, cell pellet, fat layer, or cell pellet and fat) included in the DNA extraction can affect the results (Lima et al., 2018; Sun et al., 2019). As such, some variation can be attributed to the different batches used and milk fractions included in DNA preparation.

It has previously been shown that different sequencing runs can contribute largely to microbiota composition in sequence data (de Goffau et al., 2019; Salter et al., 2014; Taponen et al., 2019). We, and others, have shown that high bacterial biomass in samples decreases the risk that sequencing results are affected by contamination (Dahlberg et al., 2019; Salter et al., 2014). In the original experiment, data on bacterial biomass in milk samples were not collected, before the infusion all quarters were considered healthy and had a low somatic cell count that is typically seen in the absence of infection. Here, the direct comparison of similarity in a created mock community between sequence runs showed a high similarity for both indexes used. These results imply that samples with high bacterial biomass are less affected by introduced biases and indicate that high similarity between sequencing runs can be accomplished.

In this study, the similarity between sequencing runs was 42% when the binary Dice index was used. These numbers are in the same range as Wen et al. (2017) presented; that similarity between technical replicates was in the range of 33%–44% when abundance not was taken into account. Schwenker et al. (2022), on the other hand, looked at variability introduced by re‐sequencing and compared that to the total variability introduced by re‐DNA‐extraction and re‐sequencing. They concluded that resequencing contributed to a major part (41%–178%) of the total variability seen.

When direct comparisons of similarity in milk samples were analyzed, within and between sequencing runs, two different similarity indexes were used. The Dice similarity index is a binary index, using the presence–absence of taxa, while the Bray Curtis similarity index, on the other hand, accounts for the abundance of taxa in the calculation. These indices were chosen to increase the robustness of the analysis.

For the comparison within the sequencing run, that is, the type 2 replicates, milk samples collected 1–2 h apart were chosen as replicates. During the first 4 h after LPS infusion, local and systemic clinical signs of inflammation were observed. At 4 h post LPS infusion, there was also an increase in somatic cell count (mainly neutrophils and macrophages) in the milk (see Johnzon et al., 2018, for details). The inflammatory response could affect the microbiota, although not likely within such a short timeframe (1–2 h). In support of this, we have recently shown that the inflammatory response to the LPS infusion did not affect the microbial composition in milk (Dahlberg et al., 2023). Further, Ganda et al. (2017) demonstrated only a minor bacterial clearance in 6 h after experimental infusion of live *E. coli* in lactating dairy cows, and also, in the wild, the estimated doubling time for *E. coli* is 15 h (Gibson et al., 2018). Both from a biological perspective and previous experiences we expected that the milk samples used in this study should be highly similar, especially in regard to their bacterial composition.

In the comparisons of the results from sequencing run 1 and 2, we observed more unique bacterial taxa per sample from run 1. However, the accumulated amount of data (i.e., reads) from the unique taxa in run 1 was much smaller than the accumulated data from the unique taxa in run 2, showing that the unique taxa in run 1 occurred in a very low abundance. This presence of low abundant taxa probably affects the similarity of samples within the sequencing run and thus explains why samples collected 1–2 h apart were less similar in run 1. Concurrently, it cannot be ruled out that the low abundant unique taxa found in sequencing run 1 could be attributed to contamination.

Other research groups have presented results that show a large variation in microbiota studies due to different batches of DNA‐extraction kits (Salter et al., 2014) or resequencing (Schwenker et al., 2022). Methods that reduce the impact of contamination (Davis et al., 2018) and working methods that “substantially reduce the contamination‐induced variability” (Moossavi et al., 2021; Wen et al., 2017) have been presented. We believe that these types of work packages can increase the repeatability of microbiota studies and that measurements of similarity between biological or technical replicates within the same sequencing run can be used as an indicator of repeatability.

We hope that the results presented here can contribute to an increased understanding of the factors that affect the results of microbiome studies and why results from different studies are not easily comparable.

## 5 CONCLUSION

We conclude that a large variability can be introduced during sample processing and sequencing in microbiota studies. This is shown through a low degree of similarity found between identical milk samples processed and sequenced (with similar but not identical lab protocols) in two separate runs. We also conclude that for milk samples collected from the same quarter within a short timeframe and analyzed in the same sequencing run, processing and sequencing can affect the level of similarity between these samples.

## AUTHOR CONTRIBUTIONS

**Josef Dahlberg**: Conceptualization (equal); Methodology (equal); Writing—original draft (lead); Writing—review and editing (equal). **Erik Pelve**: Data curation (lead); Software (lead); Writing—review and editing (equal). **Johan Dicksved**: Conceptualization (equal); Methodology (equal); Writing—review and editing (equal).

## CONFLICT OF INTEREST STATEMENT

None declared.

## ETHICS STATEMENT

None required.

## Supporting information

Supporting Information.

## Data Availability

Raw sequences data are available in the National Center for Biotechnology Information under BioProject PRJNA1013402: https://www.ncbi.nlm.nih.gov/bioproject/PRJNA1013402.

## APPENDIX A

See Figure A1.
