# Supplementary material for: Similarity in milk microbiota in replicates
Source: Microbiologyopen. 2023 Sep 30;12(5):e1383. doi: 10.1002/mbo3.1383 (PMC10542097; doi:10.1002/mbo3.1383)
Supplement: Supplementary file 1 — Supporting Information. [file MBO3-12-e1383-s001.docx]

APPENDIX

Appendix Figure A1

| ANOSIM with Bray Curtis and Bonferroni corrected p-values above the diagonal, R values below the diagonal | | | | | | | | |
| --- | --- | --- | --- | --- | --- | --- | --- | --- |
|  | run2 cow 1 | run2 cow2 | run2 cow3 | run2 cow4 | run1 cow1 | run1 cow2 | run1 cow3 | run1 cow4 |
| run2 cow 1 |  | 1 | 1 | 0.0028 | 0.0084 | 0.0028 | 0.0336 | 0.0028 |
| run2 cow2 | 0.0278 |  | 1 | 0.0224 | 0.014 | 0.0028 | 0.0028 | 0.0028 |
| run2 cow3 | 0.04222 | 0.1211 |  | 0.2268 | 0.2352 | 0.1848 | 0.322 | 0.0028 |
| run2 cow4 | 0.357 | 0.2939 | 0.2197 |  | 0.0056 | 0.0028 | 0.0028 | 0.0056 |
| run1 cow1 | 0.3678 | 0.4969 | 0.2406 | 0.5647 |  | 1 | 1 | 0.0028 |
| run1 cow2 | 0.4515 | 0.5812 | 0.269 | 0.7262 | 0.06551 |  | 1 | 0.0028 |
| run1 cow3 | 0.4072 | 0.574 | 0.2364 | 0.7029 | 0.01518 | 0.01127 |  | 0.0028 |
| run1 cow4 | 0.8299 | 0.8023 | 0.7219 | 0.4712 | 0.4798 | 0.5812 | 0.597 |  |
| ANOSIM with Dice and Bonferroni corrected p-values above the diagonal and R values below the diagonal | | | | | | | | |
|  | run2 cow 1 | run2 cow2 | run2 cow3 | run2 cow4 | run1 cow1 | run1 cow2 | run1 cow3 | run1 cow4 |
| run2 cow 1 |  | 1 | 1 | 0.0868 | 0.0196 | 0.0112 | 0.0364 | 0.0028 |
| run2 cow2 | 0.01878 |  | 1 | 0.196 | 0.0252 | 0.028 | 0.028 | 0.0084 |
| run2 cow3 | 0.03689 | 0.04162 |  | 1 | 0.1232 | 0.2492 | 0.406 | 0.0056 |
| run2 cow4 | 0.2651 | 0.2359 | 0.1453 |  | 0.0028 | 0.0028 | 0.0028 | 0.0028 |
| run1 cow1 | 0.4131 | 0.414 | 0.2693 | 0.8011 |  | 1 | 1 | 0.1848 |
| run1 cow2 | 0.3805 | 0.3597 | 0.2462 | 0.7393 | 0.09557 |  | 1 | 0.5376 |
| run1 cow3 | 0.3847 | 0.4038 | 0.205 | 0.7782 | 0.01645 | 0.06296 |  | 0.3164 |
| run1 cow4 | 0.4977 | 0.4693 | 0.4071 | 0.7198 | 0.194 | 0.1608 | 0.2005 |  |
